# Dietary, physical exercises and mental stress in a Chinese population: a cross-sectional study

**DOI:** 10.1186/s12889-021-11189-7

**Published:** 2021-06-14

**Authors:** Xiaona Li, Dan Tian, Pei Qin, Wen Guo, Jing Lu, Wenfang Zhu, Qun Zhang, Jianming Wang

**Affiliations:** 1 Health Management Center, The First Affiliated Hospital with Nanjing Medical University, Nanjing, 210029 China; 2 Department of Health Management, School of Public Health, Center for Global Health, Nanjing Medical University, Nanjing, 211166 China; 3 Department of Epidemiology, School of Public Health, Center for Global Health, Nanjing Medical University, Nanjing, 211166 China

**Keywords:** Stress, Mental health, Food frequency, Food consumption, Exercise

## Abstract

**Background:**

Mental health is affected by both genetic and environmental factors. However, previous studies have showed conflict findings about the role of lifestyle and little is known about the situation of the Chinese population. The purpose of this study was to explore the relationship between the frequency of food consumption, physical exercise condition and mental health, as well as factors related to mental stress in Chinese.

**Methods:**

We recruited 8160 residents who had health examinations in a public hospital during June 2016 to May 2018. Demographic characteristics, the frequency of food consumption, physical exercise condition and mental health status was collected by a questionnaire. We estimated the association using the odds ratio (OR) and 95% confidence interval (CI) based on Binary or ordinal logistic regression models. A classification and regression tree (CART) demonstrated the prediction of the value of a target variable based on other values.

**Results:**

The logistic regression model and classification tree model both found that the frequency of fresh vegetables or fruit and fried foods consumption and the current state of drinking alcohol were related to mental stress. The degree of mental stress reduced significantly with increasing consumption of fish (OR = 0.80, 95% CI: 0.71–0.90) and regular exercise (OR = 0.55, 95% CI:0.48–0.64) in females and increased consumption of fish (OR = 0.55, 95% CI: 0.48–0.64) and cereal crop (OR = 0.77, 95% CI: 0.68–0.89), fish (OR = 0.87, 95%CI:0.77–0.96) and regular exercise (OR = 0.61, 95%CI:0.53–0.70) in males. On the contrary, the frequency of consumption of desserts (OR = 1.43, 95% CI: 1.26–1.62) and the current of drinking alcohol (OR = 1.47, 95%CI:1.21–1.79) in females and meat (OR = 1.47, 95%CI: 1.31–1.65), pickled and smoked food (OR = 1.18, 95%CI:1.05–1.32) and the current state of drinking alcohol (OR = 1.25, 95%CI:1.12–1.40) in males were related to an increased risk of mental health.

**Conclusions:**

Our study showed that both the frequency of some food consumption and physical exercise condition were associated with mental health and affected the degree of stress, which provided novel insights into interventions.

**Supplementary Information:**

The online version contains supplementary material available at 10.1186/s12889-021-11189-7.

## Introduction

Mental stress refers to the psychological confusion or threat caused by various irritating events and adverse factors in daily life. Mental stress is strongly linked to our health both physically and mentally [1, 2]. Moderate stress has substantial, favorable effects on individual’s work or study, while chronic excessive stress may trigger severe fatigue, exhaustion even psychological disorders [3–5]. As the pace of modern life is speeding up, the negative impact of mental stress on health has gained much more attention [6, 7]. According to the World Health Organization, a quarter of people suffer from the mental stress in the world and which has been regarded as a health burden that should be highly valued [8, 9]. Despite the fact that there is no an extraordinary solution at hand to entirely avoid mental stress, people try to abate and prevent it through therapy or external intervention. And several randomized clinical trials have found that meditation programs, yoga exercise and spa therapy may reduce the adverse effects of mental stress to some extent [10–12]. Given the economic burden that these measures may create, daily diet and physical exercises could be more operable tools in reducing mental stress currently.

There is a growing body of evidences that lifestyle factors, especially diet, have beneficial effects on mental stress. Scrieks et al. observed that drinking a moderate amount of alcohol might promote the recovery of endocrine stress response [13]. Yoshikawa et al. reported a significant positive correlation between the frequency of fried food consumption and depressive symptoms among the Japanese population [14]. Furthermore, it has been found that increased intake of fruit and vegetables may help to reduce psychological pain in a follow-up study of psychological distress among middle-aged and elderly Australians [15]. Besides, moderate-intensity exercises are proved to decrease tumor necrosis factor-alpha and improve mental health [16]. However, little is known about the relationship between mental stress and the frequency of food consumption and physical exercise condition in Chinese population.

Considering the important role of diet and exercise on mental stress, the purpose of this study was to explore the relationship between the frequency of food consumption, physical exercise condition and self-reported mental health in a Chinese population and to identify factors related to mental stress for future interventions.

## Materials and methods

### Participants and procedures

Participant data were used from a dataset containing the baseline information of 8160 people who took part in a routine health examination at the Health Management Center of the First Affiliated Hospital of Nanjing Medical University during June 2016 and May 2018. In the center, all subjects must accomplish a baseline health status survey questionnaire before checkup through the WeChat platform under the instructions of specialized medical staff. The initial purpose of the baseline questionnaire is to help medical staff understand the health status and living habits of the examinee, so as to better recommend physical examination items for them. It consists of 31 items concerning demography, present symptoms, history of disease and medication, family history of illness, health behavior issues and self-reported mental stress.

However, the questions pertaining to self-rated health and mental stresses were the focus of the analysis. A total of 18 items were used in the analysis at last. (1) For diet, we collected the frequency of cereal crops, meat, fish or other aquatic products, fresh vegetables or fruits, milk or dairy products, eggs, beans, desserts, fried foods, pickled or smoked foods, and nuts consumption (days per week). (2) In terms of physical exercise, variables include frequency (days per week), exercise duration (time per day). Refer to the American College of Sports Medicine’s Guidelines for Exercise Testing and Prescription (10th ed), at least 30 min of moderate-intensity (distinguished by sweating or not during the exercise) physical activity per for 3 times per week was defined as “regular exercise”. (3) The smoking situation was divided into smoking (at least 1 cigarette per day in the past year), former smoking (quit smoking for more than 6 months), and non-smoking. (4) Drinkers were referred to those who drink ≥1 time a month, and at least 1 cup each time (1 cup contains 10 g of ethanol, such as 20 mL wine, 300 mL beer, 100 mL red wine). Non-drinkers were those who drink < 1 time per month. For drinkers, the average daily alcohol intake was calculated by the average use of wine, beer, red wine per time and the frequency (times per week). (5) To describe the mental health, participants indicated their degree of stress in life or work during the last 12 months from four categories-“not at all”, “mild”, “moderate”, and “intense”.

### Statistical analysis

We performed statistical analyses using SPSS 25 software. Continuous variables were described as the mean and standard deviation (SD), if does not conform to the normal distribution and with uneven variance they were described by the median method. Categorical variables were expressed in percentages and constituent ratios. The logistic regression model is insufficient to deal with the collinearity of variables, while the CART is not affected by the collinearity problems among variables in the analysis. The binary logistic regression model and classification and regression tree (CART) model were used to analyze factors related to mental stress. Both models took the existence of mental stress (1 = yes, 0 = no) as the dependent variable. The CART model was established by chi-squared automatic interaction detector (CHAID). The minimum sample size of parent node and child node was 100 and 50 respectively, and the maximum number of growth layers of the tree is 3 layers. Then, we explored the main influencing factors of mental stress by establishing the ordered logistic model. All significance tests were double-tailed, and the difference was statistically significant if *P* < 0.05.

## Results

After excluding 13 people with missing data on the dietary condition, 8147 subjects were included in the analysis. Ages ranged from 18 to 87. The average age was 43.1 ± 0.1 years. The ratio of male to female was 1.24:1, and the majority were married (*n* = 6751, 82.9%). The proportion of participants reporting mild, moderate, and intense stress was 17.7, 35.3, and 30.0%, respectively.

### Binary logistic regression analysis on factors related to mental stress

The binary logistic regression model was adjusted for age, marital status, smoking status and drinking alcohol circumstance. Analyses were stratified by gender groups. As shown in Table 1 and Supplementary Table 2, increased frequency of fresh vegetables or fruits (OR = 0.71, 95% CI: 0.57–0.87), fish (OR = 0.80, 95% CI: 0.67–0.95) consumption and regular exercise (OR = 0.64, 95% CI: 0.53–0.77) reduced the risk of mental stress. Contrarily, the higher frequency of fried foods (OR = 1.41, 95% CI: 1.14–1.72) intake increased the risk of mental stress. The situation of fresh vegetables or fruit consumption (OR = 0.71, 95%CI:0.58–0.87), fish (OR = 0.83, 95% CI: 0.69–0.99) consumption, and regular exercise (OR = 0.73, 95% CI: 0.59–0.90) in male were roughly parallel to female apart from meat (OR = 1.26, 95%CI:1.05–1.52) and milk (OR = 1.45, 95%CI:1.20–1.75) consumption (Table 2).

**Table 1:** Binary logistic regression analysis of the generation of mental stress on female

Terms	Mental stress, n (%)		Z	*P*	Adjusted OR (95% CI)*	P*
	No	Yes				
Cereal crop (day/week)						
0–4	129	712	23.34	< 0.01	1	0.17
5–7	648	2155			0.85 (0.68–1.07)	
Meat (day/week)						
0–4	475	1761	0.02	0.88	–	–
5–7	302	1106				
Fresh vegetables or fruits (day/week)						
0–4	168	1028	56.18	< 0.01	1	< 0.01
5–7	609	1839			0.71 (0.57–0.87)	
Fish or aquatic products (day/week)						
0–2	431	1781	11.34	< 0.01	1	0.01
2–7	346	1086			0.80 (0.67–0.95)	
Milk or dairy products (day/week)						
< 1	325	970	17.05	< 0.01	1	0.11
1–7	452	1897			1.16 (0.97–1.39)	
Eggs or their products (day/week)						
0–2	217	938	6.48	0.01	1	0.21
3–7	560	1929			0.88 (0.72–1.07)	
Legumes or their products (day/week)						
0–2	426	1677	3.37	0.07	1	0.92
3–7	351	1190			1.01 (0.85–1.21)	
Dessert (day/week)						
<1	374	1051	33.81	< 0.01	1	0.38
1–7	403	1816			1.08 (0.90–1.30)	
Fried food (day/week)						
<1	577	1649	72.10	< 0.01	1	< 0.01
1–7	200	1218			1.41 (1.14–1.72)	
Pickled or smoked food (day/week)						
<1	482	1684	2.76	0.09	1	0.68
1–7	295	1183			1.04 (0.87–1.25)	
Nuts (day/week)						
<1	365	1324	0.39	0.82	–	–
1–7	353	1307				
Regular exercise						
No	538	2333	54.14	< 0.01	1	< 0.01
Yes	239	533			0.64 (0.53–0.77)	
Marital status						
Unmarried/single	48	524	67.63	< 0.01	1	< 0.01
Married	729	2343			0.57 (0.41–0.79)	
Age (years)						
≤ 43	201	1618	228.47	< 0.01	1	< 0.01
> 43	576	1249			0.37 (0.30–0.44)	
Smoking						
None/Former	763	2797	1.11	0.29	1	0.32
Current	14	70			0.73 (0.39–1.36)	
Drinking alcohol						
None/Former	716	2541	7.98	< 0.01	1	0.12
Current	61	326			1.27 (0.94–1.73)	

*: Adjusted for marital status, age, smoking, drinking alcohol and other variables which were significant in the univariate analysis

**Table 2:** Binary logistic regression analysis of the generation of mental stress on male

Terms	Mental stress, n (%)		Z	*P*	Adjusted OR (95% CI)*	P*
	No	Yes				
Cereal crop (day/week)						
0–4	121	924	4.22	0.04	1	0.88
5–7	486	2972			1.02 (0.81–1.29)	
Meat (day/week)						
0–4	354	1943	15.00	< 0.01	1	0.01
5–7	253	1953			1.26 (1.05–1.52)	
Fresh vegetables or fruits (day/week)						
0–4	212	1887	38.51	< 0.01	1	< 0.01
5–7	395	2009			0.71 (0.58–0.87)	
Fish or aquatic products (day/week)						
0–2	307	2182	6.26	0.01	1	0.04
3–7	300	1714			0.83 (0.69–0.99)	
Milk or dairy products (day/week)						
< 1	346	1682	40.58	< 0.01	1	< 0.01
1–7	261	2214			1.45 (1.20–1.75)	
Eggs or their products (day/week)						
0–2	235	1406	1.56	0.21	–	–
3–7	372	2490				
Legumes or their products (day/week)						
0–2	326	2160	0.64	0.42	–	–
3–7	281	1736				
Dessert (day/week)						
<1	339	1796	20.02	< 0.01	1	0.67
1–7	268	2100			1.04 (0.86–1.27)	
Fried food (day/week)						
<1	405	1990	51.62	< 0.01	1	0.07
1–7	202	1906			1.22 (0.98–1.50)	
Pickled or smoked food (day/week)						
<1	364	2018	14.07	< 0.01	1	0.09
1–7	243	1878			1.18 (0.97–1.43)	
Nuts (day/week)						
<1	310	1876	2.28	0.32	–	–
1–7	263	1759				
Regular exercise						
No	436	3151	9.62	< 0.01	1	< 0.01
Yes	171	745			0.73 (0.59–0.90)	
Marital status						
Unmarried/single	52	772	26.54	< 0.01	1	
Married	555	3124			0.63 (0.46–0.85)	< 0.01
Age (years)						
≤ 43	150	2140	191.86	< 0.01	1	
> 43	457	1756			0.36 (0.29–0.45)	< 0.01
Smoking						
None/Former	351	2390	2.73	0.09	1	0.06
Current	256	1506			0.84 (0.70–1.01)	
Drinking alcohol						
None/Former	291	1793	0.78	0.38	1	0.01
Current	316	2103			1.26 (1.05–1.52)	

*: Adjusted for marital status, age, smoking, drinking alcohol and other variables which were significant in the univariate analysis

### CART analysis

**Fig. 1:**
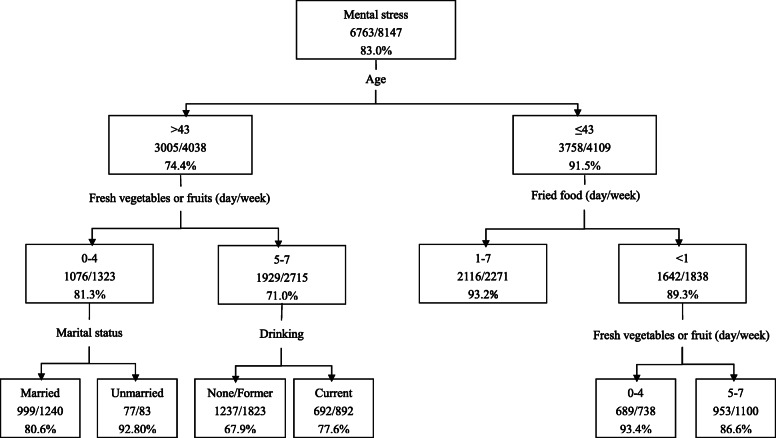
Classification and regression tree analysis on factors related to mental stress

We used the CART method to explore the role of different factors in the risk of mental stress. A total of 5 variables were included in the final model, including age, marital status, the state of drinking alcohol, the consumption of fresh vegetables or fruits, and fried foods (Fig. 1). The probability of correctly predicting the mental stress was 83.0%. The first node is age, followed by the consumption of fresh vegetables or fruit and fried foods.

### Ordinal logistic regression analysis

**Fig. 2:**
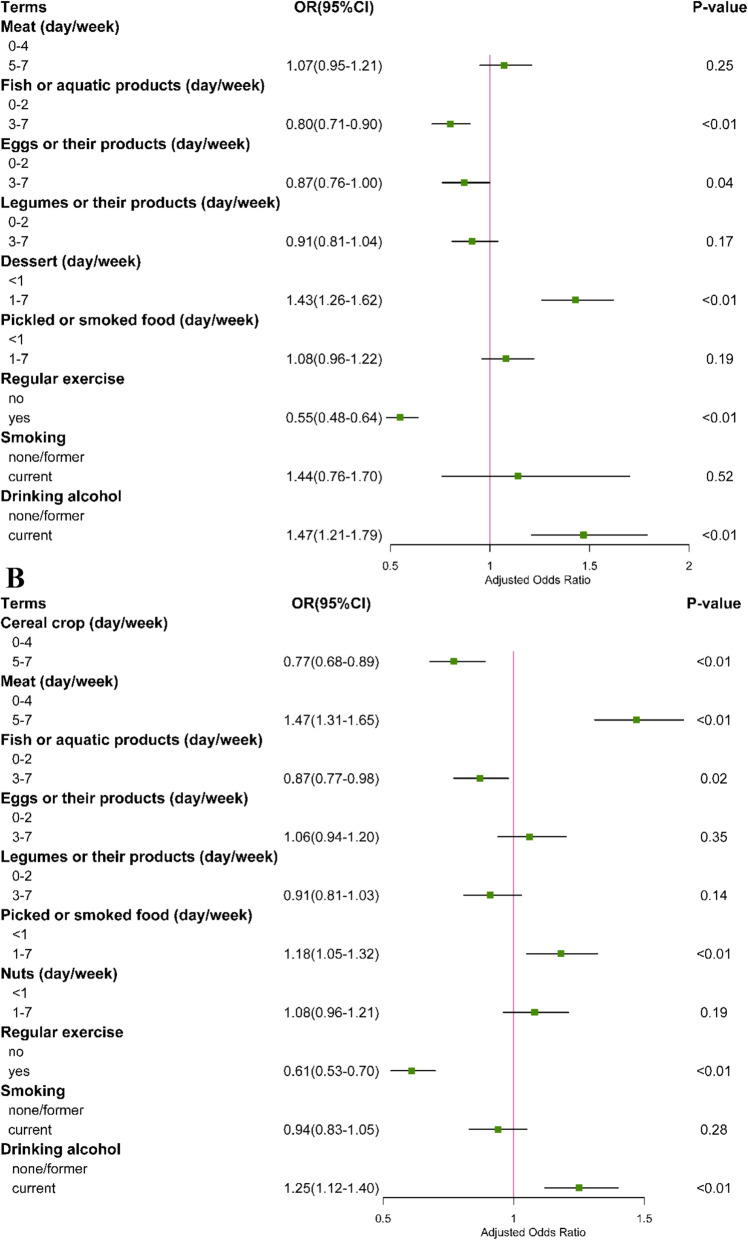
Ordinal logistic regression analysis on factors related to mental stress. **A** Ordinal logistic regression analysis for the male. **B** Ordinal logistic regression analysis for the female

We further performed an ordinal logistic regression model to explore factors related to the grade of mental stress stratified by gender. We first conducted a parallel line test, and then variables meeting the hypothesis were further analyzed in adjusted model. As shown in Fig. 2, for females, consumption of dessert 3–7 days per week (OR = 1.43, 95% CI: 1.26–1.62) and the current state of drinking alcohol (OR = 1.47, 95%CI:1.21–1.79) were related to an increased risk of mental health; the consumption of fish 3–7 days per week (OR = 0.80, 95% CI: 0.71–0.90), eggs (OR = 0.87, 95%CI:0.76–1.00) and regular exercise (OR = 0.55, 95% CI: 0.48–0.64) decreased the risk of mental stress. For males, the increased frequency of cereal crops (OR = 0.77, 95% CI: 0.68–0.89) and fish (OR = 0.87, 95% CI: 0.77–0.98) consumption and regular exercise (OR = 0.61, 95%CI:0.53–0.70) contributed to a reduced risk of mental stress, while consumption of meat 5–7 days per week (OR = 1.47, 95% CI: 1.34–1.65) and picked or smoked food 2–7 days per week (OR = 1.18, 95% CI: 1.05–1.32) and the current state of drinking alcohol (OR = 1.25, 95% CI: 1.12–1.40) could increase the degree of mental stress.

## Discussion

Psychological stress has a deleterious effect on a wide range of physical and mental health outcomes with accumulating evidence that health practices/maladaptive behaviors may mediate these relationships [17]. Considering the gender difference has been reported in lots of previous studies about the mental stress-related factors in which the stress level of women had significantly higher than that of men [18, 19], we conducted analysis after adjusting the covariate factors according to gender. The logistic regression model and classification tree model both reveal that higher consumption of fresh vegetables or fruit were associated with lower levels of mental stress. This finding is broadly consistent with many previous researches [20–23]. The protective effect of vegetables and fruit against mental stress may attribute to their plentiful antioxidant substances, which can reduce the oxidative stress and thus relieve damage to the components of neural cell [24]. Besides, the high dietary fiber intake might be link to better mental health by influencing gut microbiota [25].

In addition, mounting evidences demonstrated that fish consumption (rich in omega-3 polyunsaturated fatty acids) might improve psychological health via antioxidant action and inflammatory responses, which were consistent with precious and our studies that proper intake of fish was beneficial to mental health [26–29]. But part of studies believed that dietary intake of omega-3 fatty acids showed no association with low mood level [30]. These conflicting findings probably due to the different dietary consumption levels of omega-3 fatty acids employed in different studies (ranging from 9.6 g/day to 2.2 g/day [30, 31]) and the lack of distinction between intake of white versus fatty fish, which have varying contents of fatty acids (0.48 g/ 100 g in white fish (cod) to 5.33 g/100 g in fatty fish (mackerel) [32].

Conversely, frying can promote to generate the trans-fatty acids, and these substances accumulate in the body to increase plasma levels of inflammatory cytokines, that is, C-reactive protein, TNF-α, IL-1β and IL-6, which increase inflammation, thereby increasing the risk of mental health [33, 34]. Our research also found that more frequent fried food consumption indicates higher mental stress. Higher consumption frequency of pickled or smoked food and meat in men and dessert in women were related to an increased risk of mental stress. Picked and smoked foods are classified as unhealthy food because of the production way and increased unhealthy food intake could lead to perceived stress in young males [35, 36]. Although the meat contains high protein and essential sources of minerals such as iron and zinc which could be vitally important for mood stabilization, it also constitutes a relevant source of arachidonic acid, saturated fatty acid and cholesterol which may increase the risk of psychological depression by aggravating inflammation [37–39]. Desserts are usually full of fat and energy which are linked to increased stress while leading to obesity in women [40]. At the same time, high-stressed women seemed to prefer sweet, high-fat food more than did low-stressed women [41].

Exercise is associated with less subjective stress and buffers the effects of stress on physical and psychological health, a finding that has been observed in numerous populations from college students to older adults to veterans with post-traumatic stress disorder [42–44]. Our research also observed similar phenomenon that regular exercise exerts a positive effect on mental stress. However, not all studies found an association between stress and physical activity. The inconsistent results may be related to the differences in the methods of stress measurement, time duration or exercise intensity threshold between different studies.

Interestingly, the interaction between drinking alcohol condition and mental stress was bidirectional. Concretely, our data indicated that people who drank alcohol currently or drank more years (shown in supplementary table) had a higher probability of experiencing mental stress. And whether in cross-sectional or cohort researches, many researchers had founded that people with poor mental health were more possible to consume alcohol [45–47]. The alcohol could arouse the hypothalamic pituitary adrenal (HPA) axis and the glucocorticoid released from the end of this shaft would enter the circulation and thus activating the body’s stress response systems [48]. The effect of alcohol on generation and neurotransmission of dopamine also can not be ignored in mental stress development [49, 50].

Nevertheless, drinking alcohol ≥30 g per day in females had positive something with mental stress (shown in supplementary table), consistent with some studies which suggested that alcohol has anxiety-reducing properties and can relieve stress [49, 51]. Hence, the link between drinking alcohol and mental stress seems complex and requires to be elucidated in more detailed and specific researches. And nicotine, as the major psychoactive component, has antidepressant and anxiolytic activity in both animals and humans [52, 53], which explained the fact that the negative relationship high smoking and mental stress for males to some extent.

### Limitations and future directions

Our study had several limitations. First of all, our research was based on the data collected by the participants’ physical examination, which, as auxiliary data, has some limitations in providing complete information. For example, this data didn’t have information on extraneous factors such as weight, height, the demand of losing weight, the participants’ dietary control and exercise needs varied greatly among individuals, especially those were obese. Hence, the integrity of the data might be relatively poor. Second, the food consumption, physical exercise condition and mental stress assessment were all based on self-report, which may be affected by recalling bias. In addition, food frequency assessment was not specifically quantified, which might increase unmeasured variation, reduce precision and possibly also affects validity of the measurement. Finally, given the cross-sectional nature of the study, we were not able to assess the causality between food intake, physical exercises and mental stress. Therefore, further studies is still needed to verify the relationship between the consumption of food, physical exercise condition and mental stress via more specific and objective questionnaires and longitudinal data to confirm our results.

## Conclusions

By a large sample survey, our study demonstrated that both the frequency of some food consumption and physical exercise condition were associated with mental health and affected the degree of stress in Chinese. Corresponding lifestyle changes may have a positive effect on reducing mental stress and improving the fitness level. However, further targeted longitudinal studies are needed to examine the causal association between mental stress and lifestyle, facilitating a better understanding of the pathways through which these variables are related.

## Supplementary Information

**Additional file 1: Supplementary Table 1.** Binary logistic regression analysis of smoking situation on females. **Supplementary Table 2.** Binary logistic regression analysis of drinking alcohol situation on. **Supplementary Table 3.** Binary logistic regression analysis of smoking situation on males. **Supplementary Table 4**. Binary logistic regression analysis of drinking alcohol situation on males. **Supplementary Table 5.** Ordinal logistic regression analysis of smoking situation on females. **Supplementary Table 6.** Ordinal logistic regression analysis of drinking alcohol situation on. **Supplementary Table 7.** Ordinal logistic regression analysis of smoking situation on males. **Supplementary Table 8.** Ordinal logistic regression analysis of drinking alcohol situation on males.

## Data Availability

The datasets used and/or analyzed for the current study are available from the corresponding author on reasonable request.
