# Supplementary material for: Dietary, physical exercises and mental stress in a Chinese population: a cross-sectional study
Source: BMC Public Health. 2021 Jun 14;21:1138. doi: 10.1186/s12889-021-11189-7 (PMC8201724; doi:10.1186/s12889-021-11189-7)
Supplement: Supplementary file 1 — Additional file 1: Supplementary Table 1. Binary logistic regression analysis of smoking situation on females. Supplementary Table 2. Binary logistic regression analysis of drinking alcohol situation on. Supplementary Table 3. Binary logistic regression analysis of smoking situation on males. Supplementary Table 4. Binary logistic regression analysis of drinking alcohol situation on males. Supplementary Table 5. Ordinal logistic regression analysis of smoking situation on females. Supplementary Table 6. Ordinal logistic regression analysis of drinking alcohol situation on. Supplementary Table 7. Ordinal logistic regression analysis of smoking situation on males. Supplementary Table 8. Ordinal logistic regression analysis of drinking alcohol situation on males. [file 12889_2021_11189_MOESM1_ESM.docx]

**Supplementary Table 1.** Binary logistic regression analysis of smoking situation on females.

| Terms |  | Model 1 |  |  | Model 2 |  |
| --- | --- | --- | --- | --- | --- | --- |
|  | OR | 95%CI | *P* | OR | 95%CI | *P* |
| Smoking per day(g) |  |  |  |  |  |  |
| Low | 1 |  |  | 1 |  |  |
| High | 0.37 | 0.10-1.46 | 0.16 | 0.39 | 0.09-1.63 | 0.20 |
| Smoking time (year) | 1.02 | 0.93-1.12 | 0.69 | 1.03 | 0.93-1.13 | 0.59 |

*: Model 1: Adjusted for marital status and age; Model 2: Adjusted for marital, age, the state of smoking, exercise situation and food variables which were significant in the univariate analysis.

**Supplementary Table 2.** Binary logistic regression analysis of drinking alcohol situation on females.

| Terms |  | Model 3 |  |  | Model 4 |  |
| --- | --- | --- | --- | --- | --- | --- |
|  | OR | 95%CI | *P* | OR | 95%CI | *P* |
| Drinking dosage per day(g) |  |  |  |  |  |  |
| <15 | 1 |  |  | 1 |  |  |
| 15-30 | 0.97 | 0.40-2.34 | 0.94 | 0.84 | 0.32-2.19 | 0.72 |
| ≥30 | 0.52 | 0.24-1.13 | 0.09 | 0.42 | 0.19-0.95 | 0.04 |
| Drinking time (year) | 0.97 | 0.94-1.01 | 0.08 | 0.97 | 0.94-1.01 | 0.12 |

*: Model 3: Adjusted for marital status and age; Model 4: Adjusted for marital, age, the state of smoking, exercise situation and food variables which were significant in the univariate analysis.

**Supplementary Table 3.** Binary logistic regression analysis of smoking situation on males.

| Terms |  | Model 5 |  |  | Model 6 |  |
| --- | --- | --- | --- | --- | --- | --- |
|  | OR | 95%CI | *P* | OR | 95%CI | *P* |
| Smoking per day(g) |  |  |  |  |  |  |
| Low | 1 |  |  | 1 |  |  |
| High | 0.74 | 0.56-0.97 | 0.03 | 0.72 | 0.54-0.96 | 0.03 |
| Smoking time (year) | 0.93 | 0.92-0.95 | <0.01 | 0.94 | 0.92-0.96 | <0.01 |

*: Model 5: Adjusted for marital status and age; Model 6: Adjusted for marital, age, the state of smoking, exercise situation and food variables which were significant in the univariate analysis.

**Supplementary Table 4**. Binary logistic regression analysis of drinking alcohol situation on males.

| Terms |  | Model 7 |  |  | Model 8 |  |
| --- | --- | --- | --- | --- | --- | --- |
|  | OR | 95%CI | *P* | OR | 95%CI | *P* |
| Drinking dosage per day(g) |  |  |  |  |  |  |
| <25 | 1 |  |  | 1 |  |  |
| 25-50 | 0.85 | 0.63-1.15 | 0.29 | 0.79 | 0.58-1.08 | 0.15 |
| ≥50 | 0.77 | 0.57-1.03 | 0.08 | 0.76 | 0.56-1.03 | 0.08 |
| Drinking time (year) | 0.95 | 0.94-0.97 | <0.01 | 0.96 | 0.94-0.97 | <0.01 |

*: Model 7: Adjusted for marital status and age; Model 8: Adjusted for marital, age, the state of smoking, exercise situation and food variables which were significant in the univariate analysis.

**Supplementary Table 5.** Ordinal logistic regression analysis of smoking situation on females.

| Terms |  | Model 9 |  |  | Model 10 |  |
| --- | --- | --- | --- | --- | --- | --- |
|  | OR | 95%CI | *P* | OR | 95%CI | *P* |
| Smoking per day(g) |  |  |  |  |  |  |
| Low | 1 |  |  | 1 |  |  |
| High | 1.25 | 0.56-2.82 | 0.58 | 1.54 | 0.59-4.04 | 0.38 |
| Smoking time (year) | 1.01 | 0.95-1.08 | 0.75 | 0.98 | 0.90-1.06 | 0.55 |

*: Model 9: Adjusted for marital status and age; Model 10: Adjusted for marital, age, the state of smoking, exercise situation and food variables which were significant in the univariate analysis.

**Supplementary Table 6.** Ordinal logistic regression analysis of drinking alcohol situation on females.

| Terms |  | Model 11 |  |  | Model 12 |  |
| --- | --- | --- | --- | --- | --- | --- |
|  | OR | 95%CI | *P* | OR | 95%CI | *P* |
| Drinking dosage per day(g) |  |  |  |  |  |  |
| <15 | 1 |  |  | 1 |  |  |
| 15-30 | 0.86 | 0.41-1.70 | 0.68 | 0.78 | 0.36-1.71 | 0.54 |
| ≥30 | 1.26 | 0.73-2.17 | 0.40 | 1.20 | 0.66-2.17 | 0.55 |
| Drinking time (year) | 0.97 | 0.95-0.99 | 0.02 | 0.98 | 0.96-1.01 | 0.13 |

*: Model 11: Adjusted for marital status and age; Model 12: Adjusted for marital, age, the state of smoking, exercise situation and food variables which were significant in the univariate analysis.

**Supplementary Table 7.** Ordinal logistic regression analysis of smoking situation on males.

| Terms |  | Model 13 |  |  | Model 14 |  |
| --- | --- | --- | --- | --- | --- | --- |
|  | OR | 95%CI | *P* | OR | 95%CI | *P* |
| Smoking per day(g) |  |  |  |  |  |  |
| Low | 1 |  |  | 1 |  |  |
| High | 1.16 | 0.98-1.39 | 0.09 | 1.56 | 0.96-1.39 | 0.13 |
| Smoking time (year) | 1.04 | 1.03-1.05 | <0.01 | 1.04 | 1.03-1.06 | <0.01 |

*: Model 13: Adjusted for marital status and age; Model 14: Adjusted for marital, age, the state of smoking, exercise situation and food variables which were significant in the univariate analysis.

**Supplementary Table 8.** Ordinal logistic regression analysis of drinking alcohol situation on males.

| Terms |  | Model 15 |  |  | Model 16 |  |
| --- | --- | --- | --- | --- | --- | --- |
|  | OR | 95%CI | *P* | OR | 95%CI | *P* |
| Drinking dosage per day(g) |  |  |  |  |  |  |
| <25 | 1 |  |  | 1 |  |  |
| 25-50 | 1.21 | 0.97-1.51 | 0.10 | 1.20 | 0.95-1.51 | 0.13 |
| ≥50 | 1.17 | 0.978-1.41 | 0.09 | 1.12 | 0.92-1.37 | 0.25 |
| Drinking time (year) | 1.04 | 1.03-1.05 | <0.01 | 1.04 | 1.03-1.05 | <0.01 |

*: Model 15: Adjusted for marital status and age; Model 16: Adjusted for marital, age, the state of smoking, exercise situation and food variables which were significant in the univariate analysis.

.
